# Proliferation Tumour Marker Network (PTM-NET) for the identification of tumour region in Ki67 stained breast cancer whole slide images

**DOI:** 10.1038/s41598-019-49139-4

**Published:** 2019-09-06

**Authors:** Jesuchristopher Joseph, Martine P. Roudier, Priya Lakshmi Narayanan, Renaldas Augulis, Vidalba Rocher Ros, Alison Pritchard, Joe Gerrard, Arvydas Laurinavicius, Elizabeth A. Harrington, J. Carl Barrett, William J. Howat

**Affiliations:** 1 Molecular Pathology Group, Translational Science, AstraZeneca, Cambridge, United Kingdom; 2 Centre for Evolution and Cancer, Division of Molecular Pathology, Institute of Cancer Research London, London, United Kingdom; 3 Vilnius University, Faculty of Medicine and the National Centre of Pathology, affiliate of Vilnius University Hospital Santaros Clinics, Vilnius, Lithuania

**Keywords:** Cancer, Oncology

## Abstract

Uncontrolled proliferation is a hallmark of cancer and can be assessed by labelling breast tissue using immunohistochemistry for Ki67, a protein associated with cell proliferation. Accurate measurement of Ki67-positive tumour nuclei is of critical importance, but requires annotation of the tumour regions by a pathologist. This manual annotation process is highly subjective, time-consuming and subject to inter- and intra-annotator experience. To address this challenge, we have developed Proliferation Tumour Marker Network (PTM-NET), a deep learning model that objectively annotates the tumour regions in Ki67-labelled breast cancer digital pathology images using a convolution neural network. Our custom designed deep learning model was trained on 45 immunohistochemical Ki67-labelled whole slide images to classify tumour and non-tumour regions and was validated on 45 whole slide images from two different sources that were stained using different protocols. Our results show a Dice coefficient of 0.74, positive predictive value of 70% and negative predictive value of 88.3% against the manual ground truth annotation for the combined dataset. There were minimal differences between the images from different sources and the model was further tested in oestrogen receptor and progesterone receptor-labelled images. Finally, using an extension of the model, we could identify possible hotspot regions of high proliferation within the tumour. In the future, this approach could be useful in identifying tumour regions in biopsy samples and tissue microarray images.

## Introduction

Breast cancer is a heterogeneous disease consisting of several molecular and genetic subtypes, each with characteristic differences in clinical, biological and imaging patterns^1^. It ranks as the fifth cause of death from cancer and the most frequent cause of cancer death in women in less developed regions^2^. According to the World Health Organisation, invasive ductal carcinoma (IDC) is the most common type of breast cancer in both women and men, accounting for about 75% of all breast cancers. IDC is typically characterised by a group of malignant epithelial tumours, with invasion of adjacent tissues that have a tendency to metastasize to distant sites and that do not exhibit sufficient characteristics of a specific histological type, such as lobular or tubular carcinoma.

Tumour proliferation rate is an important prognostic biomarker^3^ with high tumour spread rates leading to worse patient outcomes. The identification of tumour proliferation rests on the identification and enumeration of mitoses in haematoxylin and eosin (H&E) stained tissues, or the use of immunohistochemistry (IHC) to label a proliferation marker such as Ki67 for proliferating cells. The Ki67 labelling index provides strong prognostic and predictive information on response to chemotherapy^4^ although it is prone to intra- and inter-observer variation^5^. To aid in the variation, digital image analysis (DIA) can be used to speed up the process and has been demonstrated to have good correlation with pathology scores^6^, but is limited by the need to identify the tumour area, which requires detailed pathology annotation. While methods exist to do this without input from a pathologist, including using a pan-tumour marker, the accurate and automated segmentation of breast cancer into tumour and non-tumour regions is challenging. The automated segmentation of tumours using Ki67 labelling is particularly problematic because Ki67 expression is not limited to the tumour and the tumour can contain Ki67-negative as well as Ki67-positive nuclei. Additionally, the segmentation of alternative IHC markers, for example oestrogen receptor (ER) and progesterone receptor (PR), requires re-training of the DIA.

Digital pathology (DP) is becoming a significant part of the pipeline in research and clinical laboratories^7^. High resolution images can be prepared for histology slides and computational tools using DIA are provided by several manufacturers, to aid in the reproducible quantification of cells and cellular expression^8^. Deep convolutional neural networks (DCNNs) have recently achieved state-of-the-art performance in various applications such as image classification^9^ and object detection^10^. Unlike the traditional hand-crafted feature approaches, deep learning represents an end-to-end feature learning, using a large amount of training data to learn high-level structural features and thereby discriminate between the classes of interest. Deep learning has been used successfully to automatically segment the epithelial and stromal regions in breast tissue using histological images stained using H&E^11^. Geert Litjens *et al*. also demonstrated that deep learning segmentation in prostate cancer gland detection significantly overlapped with pathologist annotation^12^. Therefore, the deep learning approach can serve as a good feature extractor for better data representation^13^.

In this paper, we describe a fully automated invasive breast tumour region identification system using a deep learning approach, termed Proliferation Tumour Marker Network (PTM-NET). PTM-NET can accurately detect the tumour area in IHC-stained breast cancer samples without pre-processing procedures, such as image colour unmixing or colour normalisation. In addition, following tumour identification we demonstrate that PTM-NET can identify regions of high proliferation using an activation filter map. Finally, PTM-NET can also be used to identify breast tumour regions from ER- or PR-labelled IHC without any additional training or modifications to the algorithm.

## Results

### Subjects

From the 102 samples used for analysis, the samples were split in a representative manner between training, testing and validation cohorts (Table 1). Following training, 12 images were used as test samples to examine and adjust the model using pathologist input to gain accuracy. Thereafter, the model was run directly on the validation samples.

**Table 1:** Detail of sample numbers used for training, testing and validation the model and their respective tumour percentage (mean ± standard deviation).

Tumour Percentage	Training	Testing	Validation	Average
Tumour Percentage	45.0 ± 25.9	47.9 ± 21.9	55.4 ± 22.2	49.4 ± 23.3
**Tumour Grade**	**Training**	**Testing**	**Validation**	**Total**
Low Grade (I, II)	24	5	15	44
High Grade (III)	21	7	30	58
**Total**	**45**	**12**	**45**	**102**

### PTM-NET quantitative metrics on Ki67-labelled invasive breast cancer images

**Figure 1:**
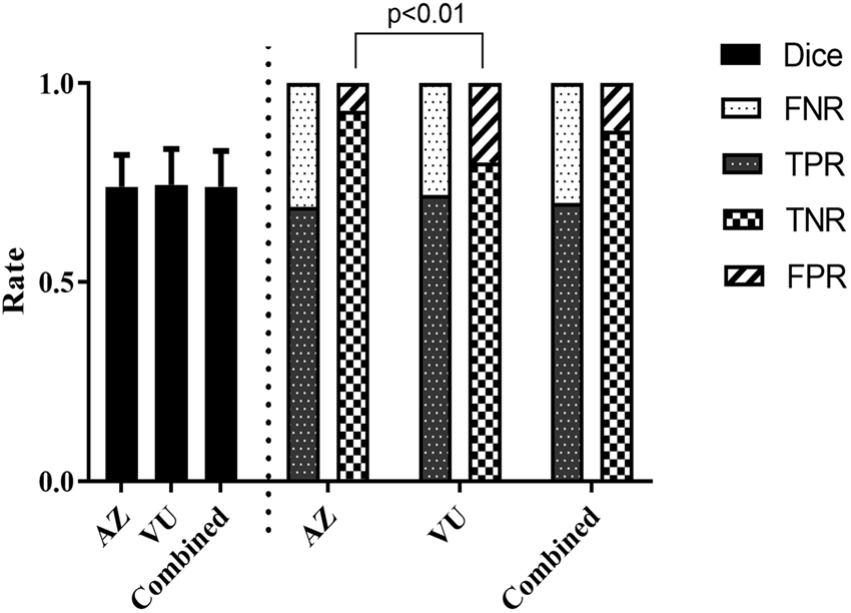
The Dice coefficient, false negative rate (FNR), true positive rate (TPR), true negative rate (TNR) and false positive rate (FPR) for the AstraZeneca (AZ), Vilnius University (VU) and Combined (AZ + VU) validation cohorts.

**Figure 2:**
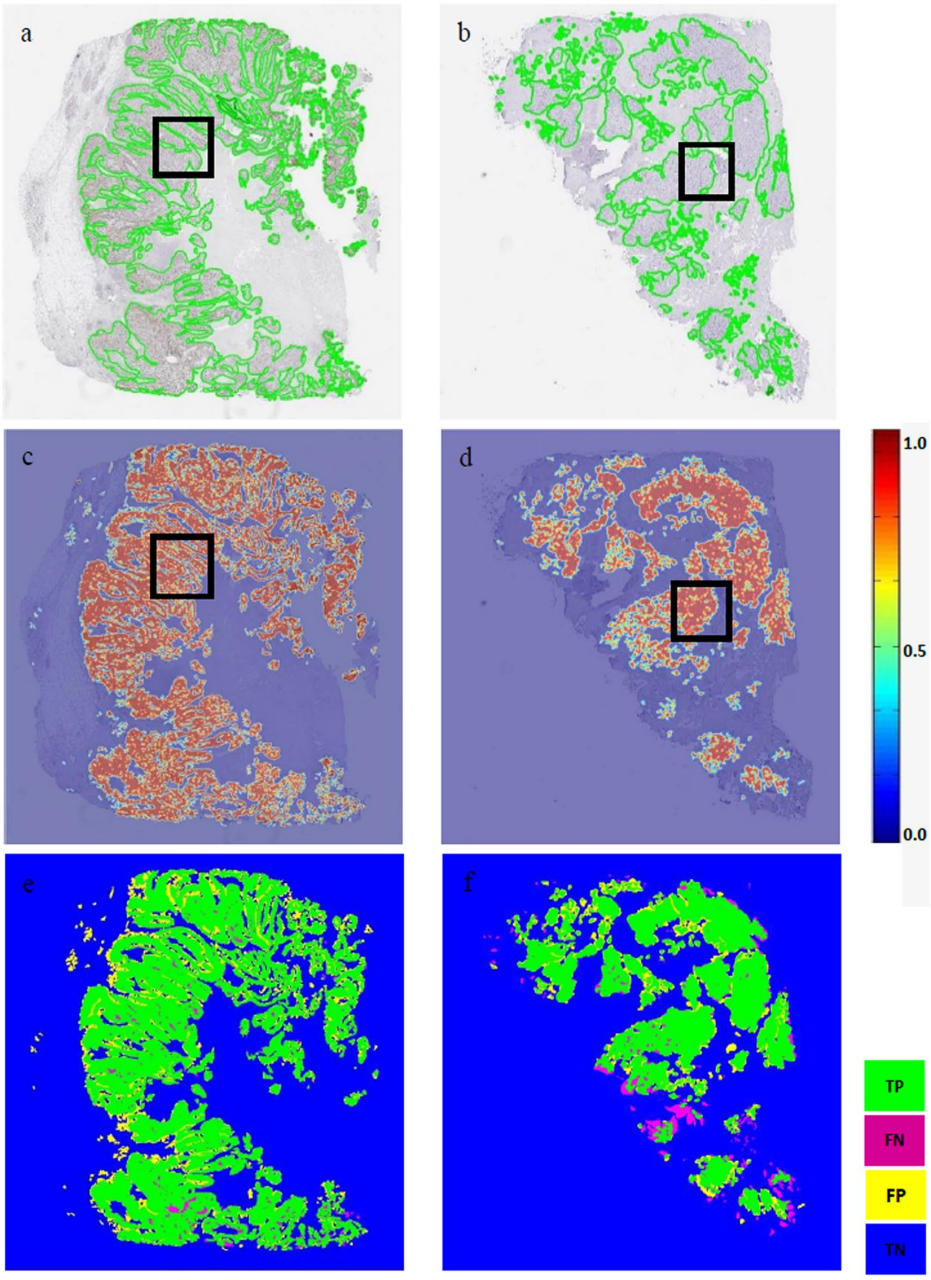
**(a**,**b)** Pathologist’s annotation (ground truth) on Ki67 whole-slide images; **(c**,**d)** the pseudo colour probability map generated by the PTM-NET classifier; and **(e**,**f)** validation results of the PTM-NET classifier in terms of true positive (TP, green), false negative (FN, pink), false positive (FP, yellow), and true negative (TN, blue) regions.

**Figure 3:**
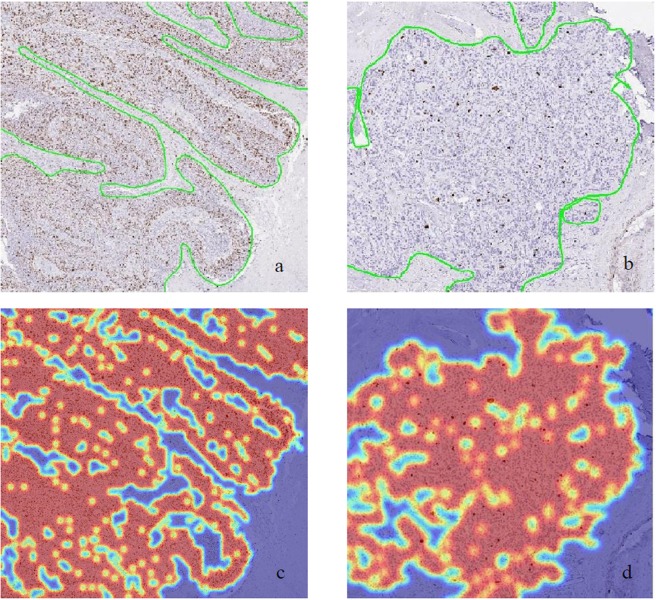
High magnification images of rectangle region marked in Fig. 2 **(a**,**b)** and **(c**,**d)** respectively.

Following PTM-NET analysis of 30 Ki67-labelled images from the AstraZeneca (AZ) validation cohort and 15 Ki67-labelled images from the Vilnius University (VU) cohort, values for the true-positive rate (TPR), true-negative rate (TNR), false-positive rate (FPR), false-negative rate (FNR), positive predictive value (PPV) and negative predictive value (NPV) were calculated, along with the Dice coefficient (DC) (Fig. 1). This demonstrated that the combined predictive value of PTM-NET for predicting non-tumour regions was high with an NPV of 88.3%, comprising TNR of 0.88 and FPR of 0.12 (Table 2). Similarly, the prediction of tumour in Ki67-labelled images was good, with a PPV of 70%, comprising TPR of 0.7 and FNR of 0.3. The Dice coefficient was 0.74. This level of accuracy can be visualised in Fig. 2, where the ground truth annotation (Fig. 2a,b) is compared to the pseudo-colour probability map (Fig. 2c,d) and the true positive (TP), false positive (FP), true negative (TN), false negative (FN) visually identified in Fig. 2e,f. Higher magnifications of Fig. 2 images are shown in Fig. 3.

**Table 2:** Performance of PTM-NET on the validation set AZ (N = 30), VU (N = 15) and Combined (AZ + VU; N = 45) cohorts.

Data set	N	TPR	TNR	FNR	FPR	PPV (%)	NPV (%)	Dice
Combined	45	0.7 ± 0.07	0.88 ± 0.07	0.3 ± 0.07	0.12 ± 0.08	70 ± 7.22	88.3 ± 7.71	0.74 ± 0.09
AZ	30	0.69 ± 0.07	0.93 ± 0.04	0.31 ± 0.06	0.07 ± 0.04	69.04 ± 6.5	92.65 ± 4.44	0.74 ± 0.08
VU	15	0.72 ± 0.08	0.80 ± 0.05*	0.29 ± 0.07	0.20 ± 0.05*	71.83 ± 8.35	79.71 ± 5.15*	0.75 ± 0.11

* denotes significant difference <0.01.

While the combined PPV, NPV and Dice coefficients were good, there were significant differences between the AZ and VU cohorts in the validation set, with a significance attached to the TNR, FPR and NPV (Table 2) with the VU values being significantly lower than the values for AZ.

There was a significant difference in accuracy of PTM-NET when combined dataset were separated by tumour grade, with a PPV of 71.7%, NPV of 86.8% and Dice coefficient of 0.76 for high grade tumours compared to a PPV of 66.4%, NPV of 91.4% and Dice coefficient of 0.7 for low grade (Grade 2 or less) tumours. When separating the combined dataset by the percentage of tumour present in the tissue sample, there was a significant difference only in NPV, with a PPV of 67.6%, NPV of 92.1% and Dice coefficient of 0.72 for tissue samples with less than 50% tumour, compared to a PPV of 71.3%, NPV of 86.3% and Dice coefficient of 0.76 for tissue samples with greater than 50% tumour (Supplementary Table 1).

### Automated detection of high proliferation region within tumour

**Figure 4:**
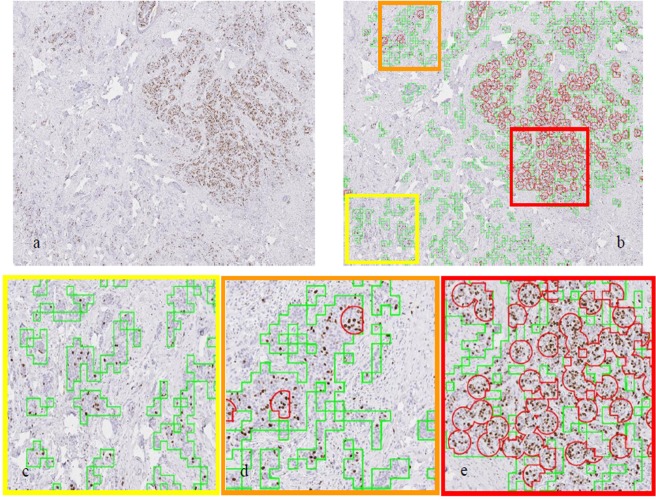
Representative example of a breast cancer whole slide image **(a)**; tumour mark-up (green) and regions of high proliferation (red) **(b)**; and **(c**–**e)** high-resolution images where low proliferation is highlighted in yellow square box, medium and high proliferation in orange and red square boxes, respectively, with reference to squares in **(b)**.

The PTM-NET model was further developed to highlight areas of Ki67 proliferation “hotspots” - to guide pathology evaluation and enumeration of the Ki67 labelling index. A representative example of an IDC with automated detection of high proliferation is shown in Fig. 4. The tumour regions were correctly identified by PTM-NET and the Ki67-labelled regions of proliferation were isolated and marked; overlays of low, medium and high proliferation are shown in (Fig. 4c–e) at a low magnification guiding to the correct region for final hot spot enumeration.

### Application of PTM-NET on breast cancer tissue with ER and PR labelling

**Figure 5:**
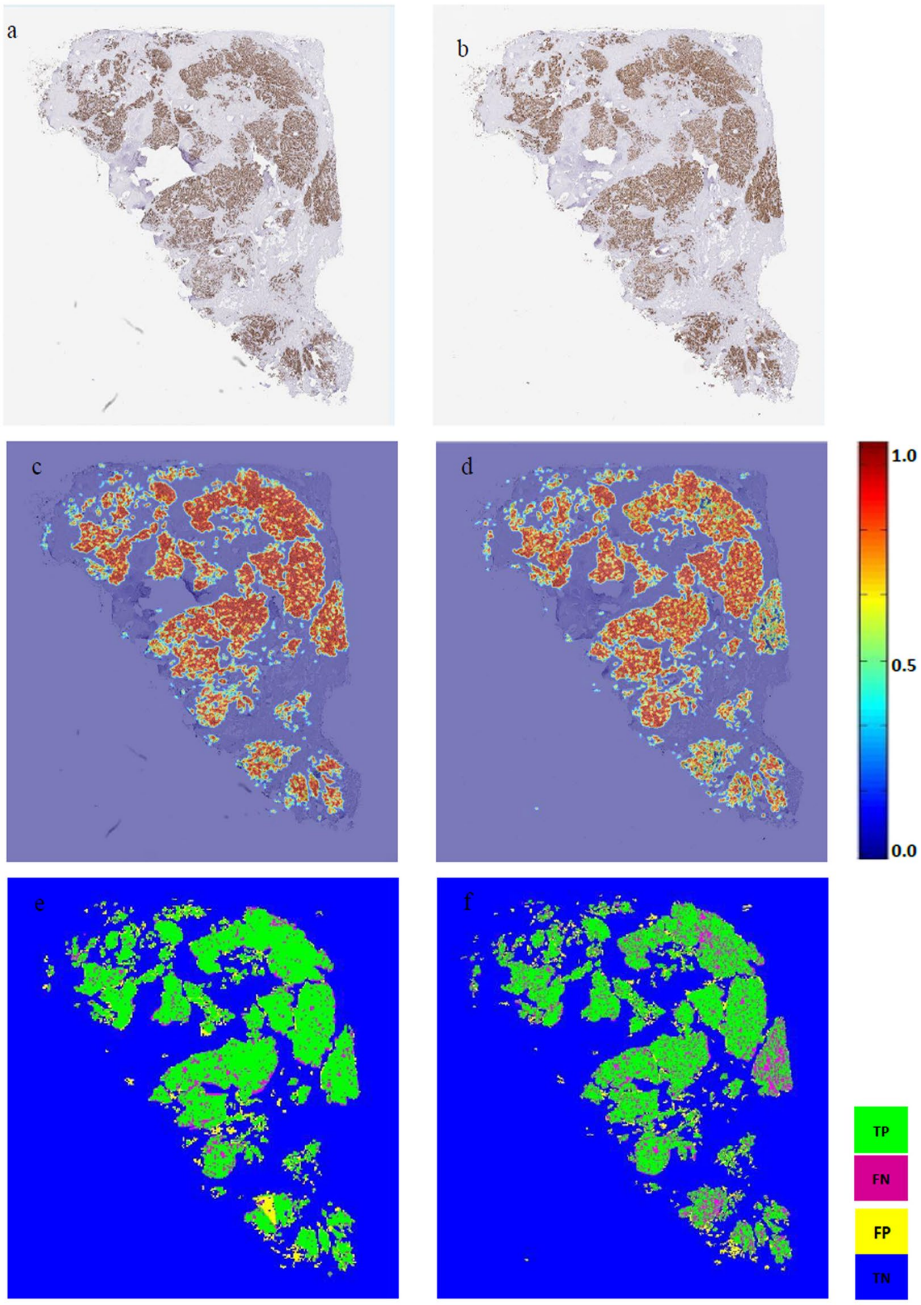
Representative examples of whole slide breast cancer images stained with oestrogen receptor (ER) or progesterone receptor (PR) and analysed using PTM-NET trained on Ki67. **(a**,**b)** Shows the original ER+ (left) and PR+ (right) images; **(c**,**d)** shows, the pseudo colour image with ER and PR +ve (red) and ER and PR −ve (blue); and **(e**,**f)** shows the validation results of the PTM-NET classifier in terms of true positive (TP, green), false negative (FN, pink), false positive (FP, yellow), and true negative (TN, blue) regions.

The accuracy of Ki67-trained PTM-NET in the segmentation of tumour and non-tumour regions in breast cancer tissues labelled with two other nuclear markers clinically relevant to breast cancer treatment, namely ER and PR, was assessed. Despite not being trained on these individual markers, Fig. 5 demonstrates that the segmentation accuracy on the ER/PR labelled tissue was good with the heatmap overlay on the tumour regions and only minimal areas of FN being identified in the PR-positive sample (Fig. 5f). The performance of PTM-NET as measured with PPV, NPV and Dice coefficient is shown in Supplementary Table 2.

### PTM-NET robustness in dealing with heterogeneous tissue images

To estimate the robustness of the PTM-NET model in predicting the tumour region, N = 5 test samples with heterogeneous architecture, staining pattern and tissue with high infiltrating pattern, were tested using a very large network architecture, VGG-NET. This comparison between the four-layer PTM-NET to the 16-layer VGG-NET were performed to demonstrate that the pathology image segmentation on both a tissue and cellular level could be faithfully achieved using a shallow four-layer network, with fewer parameters to fine tuning the network from scratch than a deep network, such as VGG-NET, that requires more parameters.

Despite the larger number of layers used for the classification, VGG-NET did not outperform PTM-NET in tumour identification. VGG-NET gave a PPV of 58.7, NPV of 91.9 and Dice coefficient of 0.69, compared to a PPV of 72.1, NPV of 93.5 and Dice coefficient of 0.77 for PTM-NET classifier, Table 3.

**Table 3:** Comparison between PTM-NET and VGG-NET in tumour classification.

Category	N	Dice	PPV (%)	NPV (%)
PTM-NET	5	0.77 ± 0.13	72.10 ± 9.68	93.51 ± 1.34
VGG-NET	5	0.69 ± 0.03	58.71 ± 5.04	91.86 ± 3.88

## Discussion

In recent years, deep learning has become the state of the art across many disciplines and is increasingly being employed in pathology. Within the all-encompassing term of deep learning in particular, the use of convolutional neural networks (CNNs) is increasing and has been employed in several applications in pathology, including the identification of invasive tumour^14^, tumour associated stroma^15^, the detection of epithelial nuclei^16^ and hotspots^17^ within H&E-stained tissue. Whilst H&E provides broad uniformity and the hue and intensity can be digitally corrected^18^, IHC provides different intensities, hues and tissue distribution depending on the antibody used and thus tissue segmentation is difficult and broadly antibody-specific.

Ki67 is an important marker in diagnostic use and has been demonstrated to predict recurrence-free survival after short-term endocrine treatment^19^. In normal and tumour regions of breast cancer tissue, Ki67 expression is non-uniform, varying across a wide range from zero to high levels of expression. Therefore, it poses a particularly difficult challenge for the training of a tissue segmentation algorithm. To overcome this challenge, we created custom CNN architecture (PTM-NET), to generate a probability map for the tumour and non-tumour regions with the exclusion of infiltrating cells, that demonstrated high segmentation accuracy, with a mean Dice value of 0.74 compared to a set of ground truth images annotated by a pathologist. Where discrepancies occurred, these were isolated small groups of tumour cells (10–30 cells) separated from the larger tumour regions. Interestingly, the NPV for the VU dataset was significantly lower than the AZ dataset, probably reflecting the lower percentage (35%) of images within the training set. These images, while using the same clone of Ki67 antibody, differ in hue and intensity of the diaminobenzidine (DAB) and haematoxylin. Surprisingly, there was a significant difference in the prediction value when separated by tumour grade, with PTM-NET showing a greater predictive value in high grade samples and samples with high tumour content (>50%). As this cannot be due to training, since the training set was intentionally split equally between grades, this must reflect the difference in tissue architecture in high grade samples and reflects the fragmentary nature of the samples with smaller percentage tumour, where the model does not perform well. However, the NPV and PPV are still good in these samples, demonstrating the applicability of the model but where further modification could improve it.

PTM-NET represents a four-layer network with two fully connected networks and input image size of 64 × 64 × 3, in contrast to Cruz-Roa *et al*. who used a three-layer network with one fully connected and an input image size of 101 × 101 × 3, generating a dice value of 0.67^14^. Whilst the increase in number of layers may be of importance, a direct comparison of the four-layer PTM-NET to the sixteen-layer VGG-NET, trained and tested on the same samples, showed that PTM-NET generated a higher tumour segmentation accuracy and significantly higher PPV, with no significant difference in segmentation of the non-tumour region. This is likely because histopathology tumour images possess colour and textural properties that are captured only using a few convolution filters and the sixteen layers of VGG-NET may add little value and comes at the expense of additional computation and higher GPU requirements.

PTM-NET is also designed for the analysis of whole slide IHC images, which is relevant for the advance of digital pathology in clinical labs worldwide that analyse a wide variety of normal tissue types and lymphocytic infiltrates, and heterogeneity of tumour and their tumour regions. In a recent advance, Xie *et al*.^20^ achieved an impressive PPV of 98%, distinguishing malignant from benign tumour on H&E stained slides, using the Inception_V3 (INV3) and Inception_resnet_V2 (IRV2) models to perform both binary and multi-class classification of the BreaKHis breast cancer image data. However, since they used transfer learning on a large set of small 700 × 460 RGB micrographs from the database, preselected by a pathologist to have tumour present, this approach does not represent a real-world scenario. Additionally, PTM-NET is designed to use IHC images and is tested on slides prepared at two different sites, with increased complexity due to differences in hue and intensity of staining both the DAB and haematoxylin channels.

The identification of hotspots as well as the quantification of IHC Ki67-positivity are of critical importance for the prognosis and the treatment of breast cancer. Counting Ki67-positive cells in the hot spot, the region of highest concentration of Ki67-positive tumour cells, is a method increasingly used in pathology^21^ where the proliferation rate is visually estimated by the pathologist over a hotspot area that could contain between 500 to 2000 cells^22,23^. This renders the identification and quantification of hotspot areas as well as the calculation of the proliferation rate complicated and time consuming. The pathologist is required to zoom out to 1X magnification (10 microns/pixel) depending on the size of the screen used and the size of the tissue sample; at this magnification many cell nuclei are smaller and will not be clearly visible. Several methods have been employed previously, including those described in a recent article by Narayanan *et al*.^24^ who used a fine-tuned VGG network based on hyper column feature maps (DeepSDCS) at a cellular resolution to detect, simultaneously segment the cells and generate seed labels used in the classification of different cell types such as stromal, lymphocytes, Ki67-positive and negative cells. Such a method relies on the accuracy of cellular segmentation before classifying into Ki67-positive/negative and tumour-positive/negative. In contrast, PTM-NET uses the reverse logic and takes the information generated from the PTM-NET classifier to segment tumour in the first instance and then analyse the Ki67-fraction within the tumour region, both of which are equally valid. Alternatively, Saha *et al*.^17^ employed a cut-off value of 15% between regions of low and high proliferation and the analysis used regions of interest on the patch images, in contrast to the PTM-NET methodology with a cut-off value of 20% combined with validation on the whole slide image. Hence this approach can be ultimately employed to minimise multiple rounds of low to high power zoom on a tissue sample to find the best sampling region. When implemented, this should improve pathologist workflow and minimise potential error.

Finally, and another demonstration of the applicability of the PTM-NET, a selection of AZ tissue samples from the Ki67 study were stained for ER and PR. While the sample number stained and analysed was small, the segmented tissue samples were visually examined and showed a high level of accuracy for the detection of tumour and non-tumour cells. Under visual examination, the only regions of discrepancy where FP or FN regions could be identified, was due to such areas not being represented in the original Ki67 training set. This discrepancy could be easily rectified and suggests that a single well-trained algorithm could be used for all IHC in breast cancer when utilising a well-designed CNN model, such as PTM-NET.

## Materials and Methods

### Tissue cohorts

Tissue blocks from 87 patients diagnosed with invasive ductal carcinoma (IDC) were acquired from commercial sources through the AstraZeneca (AZ) Biobank. From these, 45 blocks were used for training PTM-NET, 12 for the testing and subsequent re-training of the initial PTM-NET model and the remaining 30 were used for the validation. Of these 45 blocks used for training, 29 were from AZ and remaining 16 from Vilnius University (VU). Of the 45 blocks used for the validation cohort, 30 were from AZ and 15 from VU.

AZ has a governance framework and processes in place to ensure that commercial sources have appropriate patient consent and ethical approval in place for collection of the samples for research purposes including use by for-profit companies. The AZ biobank in the UK is licensed by the human tissue authority (Licence No. 12109) and has national research ethics service committee (NREC) approval as a research tissue bank (RTB) (REC No 17/NW/0207) which covers the use of the samples for this project.

### Immunohistochemical staining and image acquisition

Four, 4μm consecutive sections were taken from the formalin-fixed paraffin-embedded (FFPE) blocks and stained for H&E, Ki67, ER and PR (Table 4). For H&E, sections were stained on a Gemini H&E stainer (Thermo Fisher, UK) using Gill’s Haematoxylin (Leica, UK), and dehydrated, cleared and mounted with DPX. For immunohistochemistry (IHC), all FFPE sections were deparaffinised and rehydrated through graded alcohols before epitope retrieval using a Milestone Rapid Tissue Processor unit (Milestone, US) at high pressure in an appropriate epitope retrieval buffer (Table 4). Slides were cooled and transferred to a Labvision Autostainer (Thermo Fisher, UK) for subsequent IHC. To label Ki67, slides were peroxidase blocked with 3% H_2_O_2_ in H_2_O 20 minutes, washed in TBS-Tween and incubated in Serum Free Protein Block (Agilent, X0909) for 20 minutes. Sections were then treated with anti-Ki67 for 60 minutes and mouse Dako Envision+/HRP solution (Agilent) for 30 minutes before developing with Di-AminoBenzidine (Agilent) for 10 mins and counterstaining with Carazzi’s Haematoxylin. For oestrogen receptor (ER) and progesterone receptor (PR), the DAKO ER/PR Pharm Dx kit (Agilent) was utilised and all reagents, including antibodies were dispensed from the kit. All IHC was performed at room temperature. For Ki67-labelling performed at VU, the same antibody clone was used as for AZ, but stained using a Ventana BenchMark XT autostainers with on-board antigen retrieval and detected using the ultraView Universal DAB kit (Ventana, US)^25^.

**Table 4:** Immunohistochemical details for Ki-67 (AstraZeneca (AZ) and Vilnius University (VU)); oestrogen receptor (ER) and progesterone receptor (PR) for AZ samples only.

Antigen	Primary antibody Clone	Manufacturer/Catalogue Number	Epitope Retrieval Buffer	Epitope Retrieval Time/Temp	Primary Antibody Conc/Time
Ki67 (AZ)	MIB-1	Agilent/M7240	Sodium Citrate, pH6 (Agilent)	5 mins/110 °C	1:100, 60 minutes
ER (AZ)	1D5/ER2-123	Agilent/4071	ER/PR pharmDx Epitope Retrieval Solution (Agilent)	5 mins/120 °C	Undiluted from kit/ 30 minutes
PR (AZ)	PgR1294	Agilent/4071	ER/PR pharmDx Epitope Retrieval Solution (Agilent)	5 mins/120 °C	Undiluted from kit/30 minutes
Ki67 (VU)	MIB-1	Agilent/M2740	CC1 (Tris EDTA)	64 mins/95 °C	1:200, 32 mins

Slides were digitized using a Leica Aperio AT2 whole slide scanner at 20x objective (Leica), with a scan resolution of 0.5 µm per pixel.

### Manual annotation of training and validation samples

Using H&E slides as a reference, invasive tumour regions were annotated on the training and the validation cohort of samples by a pathologist on the Ki67-labelled images. This formed the basis of the “ground truth” for comparison of the accuracy of the algorithm. The annotations were generated using Aperio ImageScope v11.1.2 on the down sampled image with the apparent magnification of x10 which contained sufficient contextual information to train the PTM-NET model. All selected training samples contained distinct examples of tumour, and non-tumour, and captured different expression of Ki67 within IDC (Supplementary Fig. 1).

### Proliferation tumour marker network (PTM-NET)

**Figure 6:**
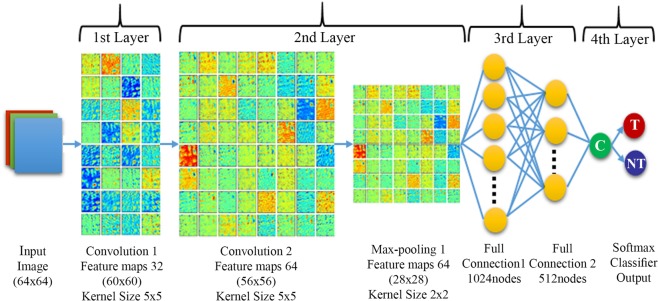
Architecture of the 4-layer PTM-NET.

PTM-NET is a custom designed deep learning model whose architecture is designed based on a convolution neural network (CNN) of four layers as shown in Fig. 6. The first layer comprises a convolution layer with 32 filters, kernel size of 5 × 5 and stride of 1. The second layer has a convolution layer with 64 filters, kernel size of 5 × 5 and stride of 1 leading to a max-pooling layer of kernel size 2 × 2. The third layer consists of two fully connected layers with 1024 and 512 neurons, respectively, and the final layer is a softmax classifier consisting of two labels as possible output, one for tumour and the other for non-tumour.

The mean normalised input RGB (red, green and blue, three channel) image patches of size W × H (W = width; H = Height) was convolved to convolution layers with N (N = Numkernels; Convolution1 = 32, Convolution2 = 64) filter banks with kernel size of M × M (ex: 5 × 5; 2 × 2) to generate the network activation map. For each input image patch, after the convolution process, the feature map will be equal to the number of filter banks. Dependant on the size of the input image, kernel size and the stride, the size of the output feature map can be estimated using:

$${\rm{NumKernels}}\times \frac{{\rm{W}}+2\times {\rm{pad}}-{\rm{M}}}{{\rm{stride}}}\times \frac{{\rm{H}}+2\times {\rm{pad}}-{\rm{M}}}{{\rm{stride}}}$$

To train the CNN model, image patches of size 64 × 64 pixel (W × H) were generated using a sliding window technique within the annotated region. The patches were extracted with a pixel resolution of approximately 0.5 µm per pixel at 20x objective. The training patches were categorised as tumour (positive examples) and non-tumour (negative examples) and labelled according to the supervised training. The convolution process was followed by a max pooling operation which reduces the feature map size by half as the kernel size was 2 × 2. In addition to performing down sampling of the feature map, max pooling provides the feature map resistance to the translation-invariant. The ReLU activation used in the study is of the form *f* (*x*) = max (0, *x*)^26^.

Dropout is usually performed on the full connection layer which excludes the non-active neurons during each training iteration^27^. Using this procedure significantly improved the training time and reduced the computational time required when training a model for classification purposes. A stochastic gradient descent algorithm was used to update the network weights to minimise the loss function. The softmax has the property of a smooth gradient so that the back-propagated error is not subject to discontinuities, allowing for easier training. The trained SMC classifier yielded an output based on equation (1).

1 $$\begin{array}{ll}\sigma ({\boldsymbol{z}})j=\frac{{e}^{{z}_{j}}}{{\sum }_{k=1}^{K}{e}^{{z}_{k}}} & {\rm{for}}\,j=1,\,\ldots \,,\,K\end{array}$$

were σ is logistic function, *z*_*j*_ is a net input and *K* is probability values.

**Figure 7:**
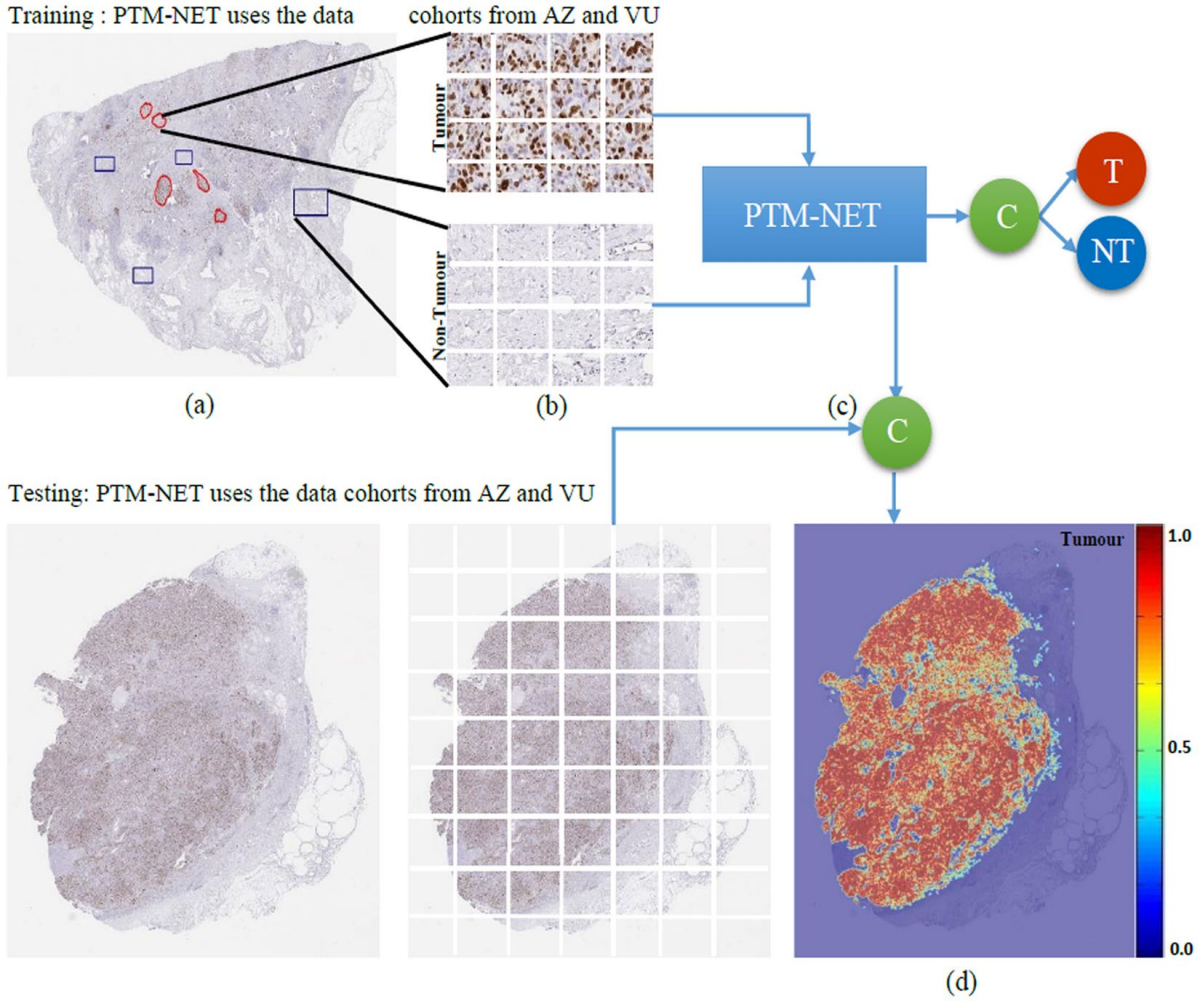
Pipeline for training the PTM-NET model to classify tumour and non-tumour **(a)** pathologist annotation, **(b)** training samples, **(c)** PTM-NET model for training and testing and **(d)** pseudo-colour probability map as tumour (red) and non-tumour (blue).

The PTM-NET classifier was trained using images from the AZ and VU datasets that had been annotated into tumour and non-tumour regions by a pathologist. From this, 222,716 patches were extracted, of which 117,218 were from the tumour class and 105,498 were from the non-tumour class (Fig. 7). Data augmentation was used in this study. The weights and bias of the CNN were randomly initialized. The CNN parameters were updated and optimized during the training process using the stochastic gradient descent algorithm. The learning rate was set at 0.001 with the momentum of 0.9. The classifier was trained for 15 epochs with a batch size of 25 and a dropout layer was inserted after the second FC layer to avoid over-fitting. After rigorous experimentation, it was discovered that dropout ratio of 0.5 provided the best result. ReLU was employed after each convolutional layer to speed up the computing time. The Deep learning pipeline was developed using an Intel® Core™ i7-8850H @ 2.60 GHz, Windows 10, 64GB memory workstation using Tensor flow and Keras library.

To test the trained PTM-NET to identify the tumour regions on whole slide images, overlapping 64 × 64 image tiles were segmented from twelve whole slide images and the model was applied to predict the probability map. To speed up the process of probability prediction any non-tissue regions were avoided using a suitable threshold operation. Areas of high probability (p > 0.75) of tumour were marked in red and low probability in orange (0.65 < p < 0.75) or yellow (0.55 < p < 0.65). The non-tumour regions were marked in blue as shown in Fig. 2. A pathologist reviewed the tumour probability map generated by the PTM-NET on the test set and marked the FP and FN regions in the probability map. These false regions were added to the training samples to retrain the model to learn the features from these tissue regions.

As part of the PTM-NET model, inflammatory infiltrate was specifically excluded. For this, an infiltrate model was trained with annotated down-sampled images from three representative breast cancer samples, classifying into positive probability of lymphocytic infiltrate and zero probability. The output from this model identifying the infiltrate region is shown in the Supplementary Fig. 2.

### Validation metrics

We validated the accuracy of the PTM-NET classifier in whole slide images by comparing the predictions of tumour regions made by the PTM-NET in the validation data set against the corresponding ground-truth regions annotated by a pathologist on the same 45 images, included slides from both the AZ and VU cohorts. A quantitative evaluation was performed by measuring the Dice coefficient, positive predictive value (PPV), negative predictive value (PPV), true positive rate (TPR), true negative rate (TNR), false positive rate (FPR) and false negative rate for (FNR) across all 45 validation slides. In addition, the mean and standard deviation performance measures were calculated for each validation data cohort.

### VGG-NET comparison

A transfer learning approach was adopted to train the VGG-NET^28^ (VGG16) used in this study. Top layer weights were initialized from the ImageNet^9^. Two fully connected layers followed by a softmax layer were added to the top layer with random weights initialization to achieve binary classification. To train the VGG-NET to perform tumour classification, the total samples were divided into seventy percent training and thirty percent testing data. Dice, PPV and NPV were estimated to determine the performance of the trained model versus the ground truth manual annotation.

### Whole slide breast tumour detection and high proliferation identification system

**Figure 8:**
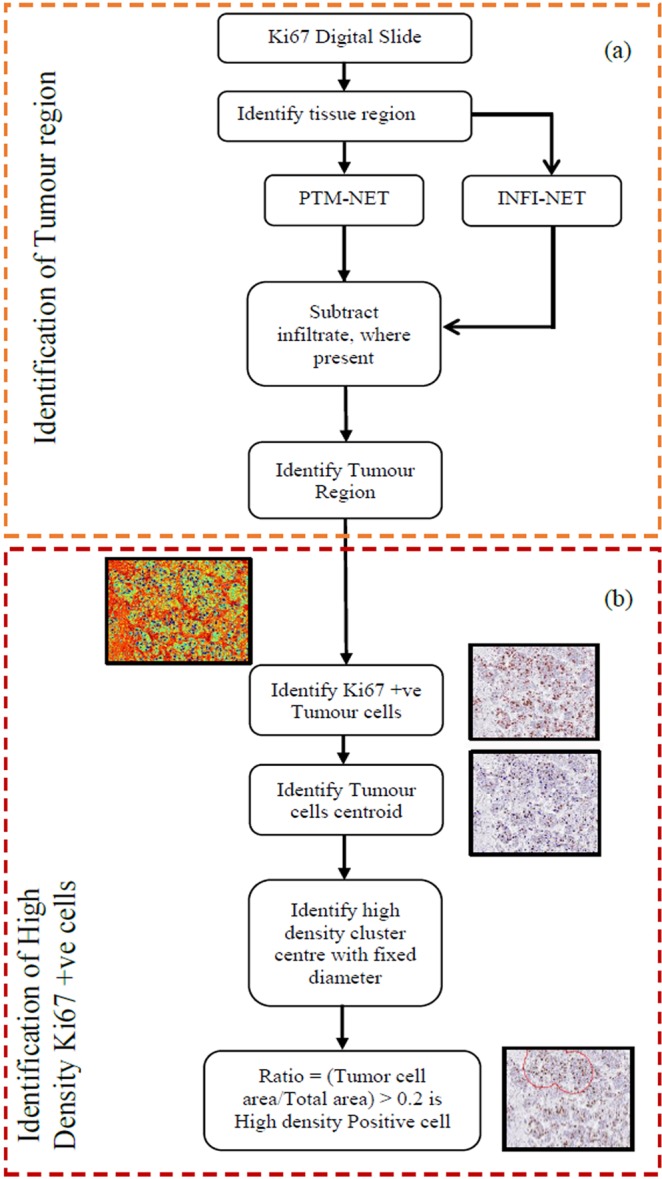
Overall workflow of PTM-NET for **(a)** identification of the tumour region and (**b)** identification of high density Ki67 +ve tumour cells.

The overall workflow of the breast tumour detection and high proliferation identification system is shown in Fig. 8. Briefly, tissue regions were identified by measurement of the patch mean intensity (MI) of a 64 × 64 pixel size, were cropped from the whole slide image using a sliding window technique. Regions exhibiting an MI of <235 were considered as tissue and the remaining classified as background. These tissue regions were then fed into the PTM-NET and INFI-NET classifiers to identify tumour and lymphocytic infiltrate in the tissue regions.

From these restricted tumour regions, DAB-stained cells were extracted using threshold and morphological operations from the activation map as a binary image and overlaid on the original image for visual control. The subsequent binary mask of the DAB cells was used to generate the centroid (blue) for every identified cell and cluster centres (red) were identified using a subtractive clustering method. A circle was drawn with a diameter of 100 pixels for every cluster centre and used to estimate the Ki67 ratio (DAB area to the total area) within the circle to estimate if there was a region of high proliferation. Finally, on the original image, the regions where the Ki67 ratio was greater than 20 percent were annotated in red circle.

### Accuracy of PTM-NET to other nuclear markers

We validated the accuracy of the PTM-NET classifier in both ER and PR-labelled breast cancer samples, choosing three ER or PR-labelled whole slide images from the validation cohort and comparing the ground-truth annotation of the Ki-67 regions to the PTM-NET evaluation of the ER or PR-labelled images.

## Supplementary information

Supplementary Figures and Tables
