# Supplementary material for: Proliferation Tumour Marker Network (PTM-NET) for the identification of tumour region in Ki67 stained breast cancer whole slide images
Source: Sci Rep. 2019 Sep 6;9:12845. doi: 10.1038/s41598-019-49139-4 (PMC6731323; doi:10.1038/s41598-019-49139-4)
Supplement: Supplementary file 1 — Supplementary Figures and Tables [file 41598_2019_49139_MOESM1_ESM.pdf]

SUPPLEMENTARY MATERIALS

Proliferative Tumour Marker Network (PTM-NET) for the identification of tumour region in Ki67 stained breast cancer whole slide images

*Jesuchristopher Joseph[1], Martine P. Roudier[1], Priya Lakshmi Narayanan[2], Renaldas Augulis[3], Vidalba Rocher Ros[1], Alison Pritchard[1], Joe Gerrard[1], Arvydas Laurinavicius[3], Elizabeth A. Harrington[1], J. Carl Barrett[1] and William J. Howat[1]

SUPPLEMENTARY FIGURE

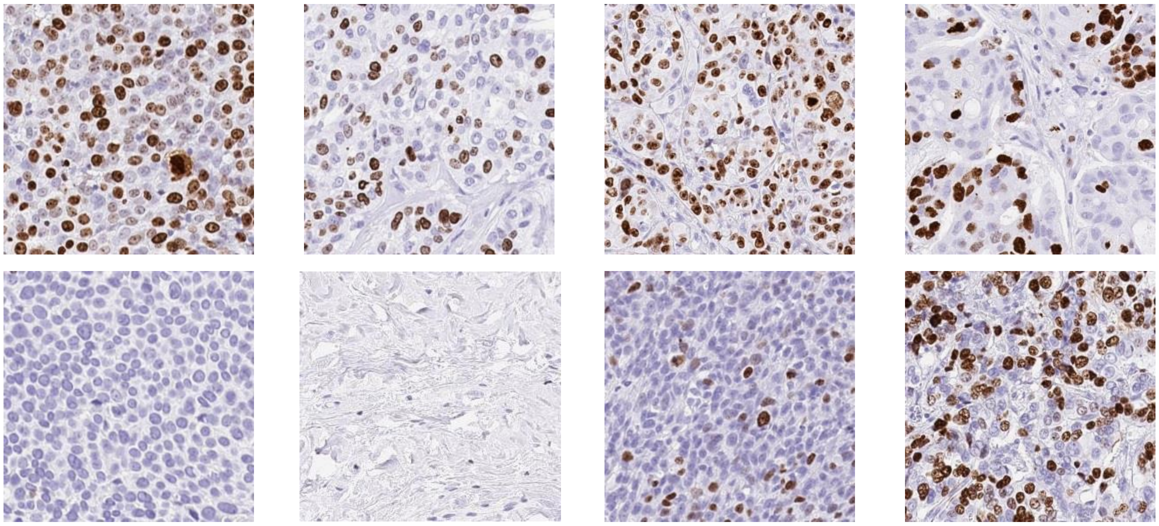

Supplementary Figure 1. Examples of Ki67-labelled microscopic images demonstrating the heterogeneity of staining across the tumour and non-tumour samples used for training PTM-NET.

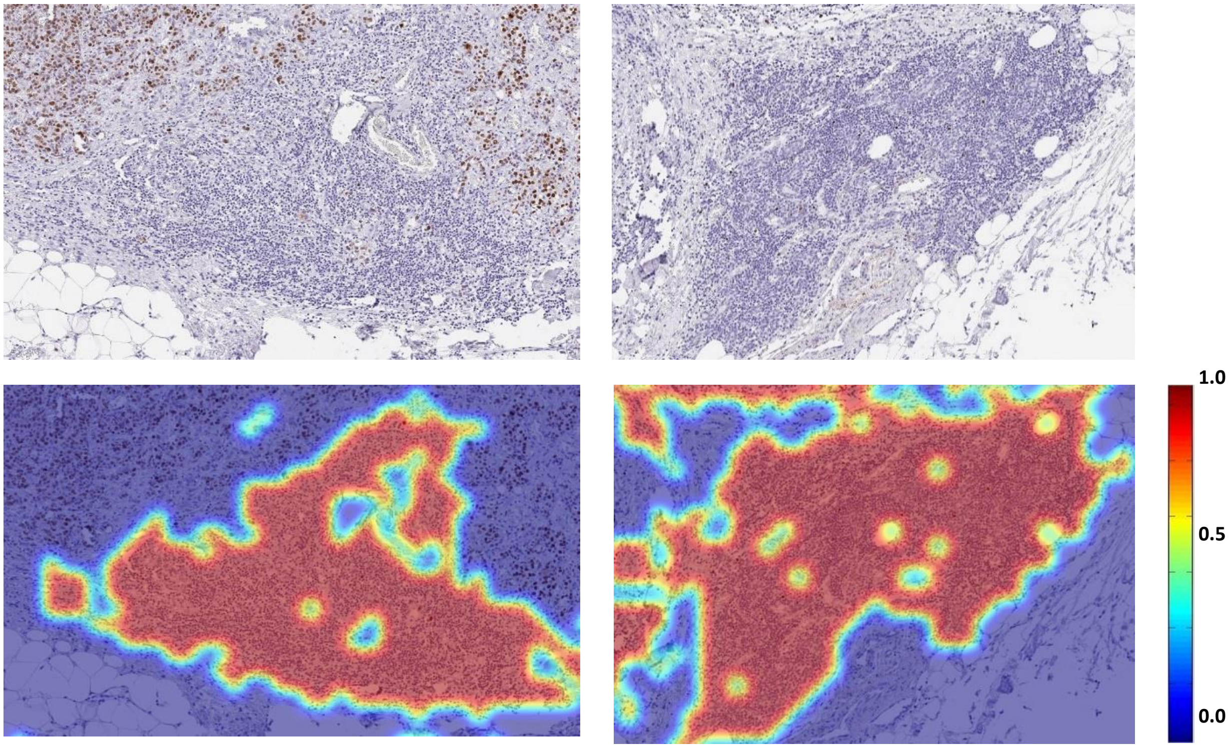

Supplementary Figure 2. Representative examples of Ki67-labelled breast cancer images with infiltrate from two different samples (top row). The bottom row shows the high probability infiltrate region marked in red and less probability in orange and yellow color.

SUPPLEMENTARY TABLE

Supplementary Table 1. Performance measure of the PTM-NET on the combined AZ and VU cohorts compared with grade and percentage of tumour. * denotes significant difference <0.05.

Data set	N	TPR	TNR	FNR	FPR	PPV (%)	NPV (%)	Dice
**High Grade (Grade 3)**	30	0.72±0.07	0.87±0.08	0.28±0.07	0.13±0.08	71.7±7.6	86.8±8.13	0.76±0.09
**Low Grade (Grade</=2)**	15	0.66±0.05*	0.91±0.06*	0.33±0.05*	0.08±0.06*	66.4±5*	91.4±5.89*	0.7±0.08*
**High % Tumour (>50%)**	29	0.71±0.08	0.86±0.07*	0.29±0.08	0.14±0.07*	71.3±8.1	86.3±7.5*	0.76±0.1
**Low % Tumour (<50%)**	16	0.68±0.05	0.92±0.07	0.32±0.04	0.08±0.07	67.6±4.64	92.1±6.72	0.72±0.07

Supplementary Table 2: Performance measure of the PTM-NET on the oestrogen receptor (ER) and progesterone receptor (PR)- labelled samples.

Category	N	Dice	PPV (%)	NPV (%)
**ER**	3	0.72±0.04	64.53±3.49	92.89±3.48
**PR**	3	0.74±0.01	65.37±0.91	93.83±2.32
